# How cells overcome egoism

**DOI:** 10.1007/s00709-025-02151-0

**Published:** 2025-12-17

**Authors:** Peter Nick

**Affiliations:** Joseph Gottlieb Kölreuter Institute für Plant Sciences, Karlsruhe Institute of Technology, Karlsruhe, Germany

Competition for limited resources has been considered as the driving force for evolution. The idea can be traced back to almost two and a half millenia ago: “There is enmity between such animals as dwell in the same localities or subsist on the food. If the means of subsistence run short, creatures of like kind will fight together” (Aristoteles 340 BC, in Thompson 1913). It is evident that this principle must have governed the interactions between the unicellular organisms that later gave rise to differentiated multicellular forms of life. The fundamental split between propagation and differentiation came with the cost that the differentiating cells were doomed to death. In his essay *Über die Dauer des Lebens* (On Lifespan), Weismann (1881) stated that death is not an inevitable consequence of multicellularity, since it would be conceivable that the somatic cells just loose totipotency, but still continue to generate cells of their own kind. He then continues that mortality might have been developed actively, as a way to adapt to the external conditions. By developing the capacity to sacrifice the own survival for the survival of the propagating cells, somatic cells enabled multicellularity, opening up novel pathways to cope with the environment. In other words: there was a shift from cellular egoism to altruistic behaviour. Three contributions to the current issue address different facets of this astonishing phenomenon.

In their comprehensive review, Doronina and Lazareva (2026**)**, shift programmed cell death (PCD) of plants into a broad evolutionary context. They start with a tour through different forms of PCD, beginning with bacteria and protists. At first sight, one would not expect PCD in unicellular organisms, where cells compete for limited resources. However, the authors describe numerous examples, where the survival of few cells in a population is secured by altruistic sacrifice of other cells to provide the resources. After describing the salient features of PCD in fungi, invertebrates, and vertebrates, they arrive at their main topic, plant PCD, discussing not only the cellular events, but also the molecular players. Metacaspases arose early in evolution and were passed on several times by horizontal gene transfer, they were apparently lost in animals and then replaced by the caspases, derivatives of the older paracaspases. To understand, how the players and their complex interaction could have arisen in evolution, the authors revisit the “Original Sin” hypothesis (Ameisen 2002) proposing that PCD was already acting in the Last Universal Common Ancestor of Life (LUCA). The different players that had to assemble to give birth to such a complex phenomenon as PCD, often play additional functions in different contexts that are not linked with self-destruction. As it should be the case for a fruitful review, the authors transcend mere collation and structuring of information by addressing open questions and suggesting lines for future research. They make a strong stand for an evolutionary perspective, proposing following up the players of PCD across different life forms and also focussing on their additional functions outside of cell death. During their excursion through the evolution of cell death, they also elaborate on the different versions of this phenomenon and suggest that the two accepted forms of PCD in plants might be due to gaps in nomenclature, because details of function and mechanisms have been ignored. A third of their suggestions is also to focus on regulatory networks, described for the model plant Arabidopsis thaliana as “deathosome”.

In many organisms, PCD is activated in the context of cell proliferation, basically as a kind of quality control ensuring organismic integrity by eliminating damaged or mutated cells. The stringency of this control is expected to be accentuated when resources become limiting. The contribution by Ballesteros-Barrera et al. (2026) address this aspect for the root meristem of the model plant *Arabidopsis thaliana*. By their proliferation, the meristem delivers the cells that by their expansion will drive the subsequent growth of the root system. Since the meristematic cells will progressively differentiate, they need to be replenished by occasional divisions of stem cells in the so-called quiescent centre. It is evident that the divisions of both, stem cells and meristematic cells, need to be synchronised with the external conditions. A central complex for eukaryotic transcription, MEDIATOR, plays a central role for this synchronisation. In fact, a loss-of-function mutant for a component of this complex, MEDIATOR 18, suffers from progressive deterioration of the meristem. Interestingly, the penetration of this mutation can be modulated by conditions. For instance, cultivation in the dark, constraining energy resources, mitigates the phenotype. The authors decided to use phosphate starvation as signal. Phosphate, as component of nucleotides, is essential for cell proliferation and is often a limiting factor for plant growth. In response to phosphate starvation, root architecture is remodelled, by reducing elongation of the main root and stimulating the growth of lateral organs, such that the root system can forage the environment for other sources of Phosphorous. Surprisingly, phosphate depletion, in itself impairing meristematic proliferation, and mutation of MEDIATOR 18 subunit, in itself impairing meristematic proliferation as well, are mutually mitigating. Searching for an explanation, authors collect evidence for a model, where phosphate deficiency can activate a pathway processing DNA damage leading to cell-cycle arrest. They suggest that MED18, on the other hand, responds to phosphate starvation by elevated uptake of iron, leading to elevated oxidative states, and restraining accumulation of the growth hormone auxin. This would, by a second pathway, converge on cell cycle regulation. When cell death is a tool for quality control, phosphate depletion should mitigate the impact of genotoxic stress. The authors test this implication by treatment with zeocine and can confirm that, indeed, phosphate starvation helps to sustain genetic integrity of rapidly cycling cells.

Altruistic behaviour requires that the needs of the others can be perceived. Thus, PCD is intimately linked with cell-cell communication. This aspect differs fundamentally between plant and animal cells, because plant cells sustain cytoplasmic continuity through the plasmodesmata even to an extent that ER and actin filaments are shared between neighbouring cells. This aspect is so fundamental that this journal dedicated a special issue on plasmodesmata (Heinlein et al. 2011). To use a metaphor: a plant could be likened to a castle, where the doors between the numerous rooms are kept open, such that a passer-by can go from any room to whereever he wants without the need to pass through a barrier. Of course, this metaphor is oversimplified, because there are different types of doors that can be passed by certain molecules, but not by others, and this passage is subject to dynamic and complex regulation. In another comprehensive review, Lv et al. (2026) discuss different methodologies to study plasmodesmata in action. After a brief overview on plasmodesmata and channels deriving from them they start off with electron microscopy, connecting classic studies with novel developments that extend into the three-dimensional topology, such as scanning electron microscopy combined to serial stacks of ultrathin sections. They continue with fluorescence-optical techniques, including novel approaches breaching the resolution limit, such as structured illumination microscopy. It is a special merit of this review that the authors not only demonstrate the power of microscopy in studying the structural details of plasmodesmata and their derivatives, but also in exploring their function, for instance by determining size exclusion limits, specificities of transport, or dynamic changes in spatial distribution of those channels. A second merit is the critical discussion of the respective limitations of those methods. While cell biology is dealing with the spatiality of molecules and organelles, there is also a more physiological side to it. Structures are more than a static skeleton, they guide and drive dynamic events that, in turn, act back on cellular structures. To get a fuller picture, our cell biology toolbox needs to be developed towards visualising activities, not just structures.

It seems that the ability to overcome cellular egoism was a prerequisite for multicellularity. This means that altruistic behaviour had to develop first, before cells could team up to form a common organism. This shifts cell-cell communication into the focus, a task requiring a shift of paradigm in cell biology. While structural and molecular aspects are needed to understand cell communication, they are certainly not sufficient, but need to be complemented by approaches to follow the interactions between cells, especially under conditions, where the shift from competition to cooperation. We might need a sociology of cell death to understand the evolution of multicellular life forms.
